# Dual Prosthetic Heart Valve Presented with Chest Pain: A Case Report of Coronary Thromboembolism

**DOI:** 10.1155/2015/895473

**Published:** 2015-02-16

**Authors:** Supakanya Wongrakpanich, Natanong Thamcharoen, Pakawat Chongsathidkiet, Sarawut Siwamogsatham

**Affiliations:** ^1^Department of Medicine, Albert Einstein Medical Center, Philadelphia, PA 19141, USA; ^2^Department of Medicine, Bassett Medical Center and Columbia University College of Physicians and Surgeons, Cooperstown, NY 13326, USA; ^3^Division of Neurosurgery, Department of Surgery, Duke University Medical Center, Durham, NC 27710, USA; ^4^Division of Hospital Medicine, Department of Medicine, Faculty of Medicine, Chulalongkorn University and King Chulalongkorn Memorial Hospital, Thai Red Cross Society, Bangkok 10330, Thailand

## Abstract

Coronary embolism from a prosthetic heart valve is a rare but remarkable cause of acute coronary syndrome. There is no definite management of an entity like this. Here we report a case of 54-year-old male with a history of rheumatic heart disease with dual prosthetic heart valve and atrial fibrillation who developed chest pain from acute myocardial infarction. The laboratory values showed inadequate anticoagulation. Cardiac catheterization and thrombectomy with the aspiration catheter were chosen to be the treatment for this patient, and it showed satisfactory outcome.

## 1. Introduction

Apart from coronary artery spasm, coronary embolism is a rare but distinct cause of acute coronary syndrome in normal coronary arteries [1]. Coronary embolism is responsible for 1%–17% of coronary occlusion in autopsy cases [2]. The underlying reason for the small incidence is the size of emboli, the small caliber of the coronary arteries, location and angle of coronary arteries at the root of the aorta, and the least coronary flow during systole [2, 3].

The causes of coronary emboli include bacterial endocarditis [4] (which is the most common cause and responsible for more than half of the cases [2, 5]), nonbacterial endocarditis, prosthetic heart valve, coronary artery disease, heart valve surgery [6], cardiomyopathy, paradoxical embolism, aortic thrombus, cardiac myxoma, and pulmonary vein thrombosis [3, 5, 7]. We are reporting a case with dual prosthetic heart valves representing one of the causes of coronary embolism.

## 2. Case Presentation

A 54-year-old Thai male presented with a constant, nonradiating, heavy chest pain at the middle of his chest that has lasted for 3 hours prior to admission. He rated the severity of the pain as 8 on a 10-point scale. He also had sweating and palpitation but there was no orthopnea or paroxysmal nocturnal dyspnea. He has a medical history of rheumatic heart disease (mitral stenosis, mitral regurgitation, and aortic regurgitation) and underwent ball-caged metallic valve replacement at both mitral and aortic valve areas approximately 20 years earlier. His last echocardiogram was done 10 months ago and showed a dilated left atrium and left ventricle with moderately impaired left ventricle systolic function with global hypokinesia (left ventricular ejection fraction 42%). Also, there was an increased gradient across the atrioventricular prosthesis, normal mitral valve prosthesis function, no paravalvular leakage, no rocking motion, no intracardiac thrombus, and no pericardial effusion. He also has a history of left embolic stroke 15 years ago. During that time, he presented with right facial palsy and dysarthria. He had now fully recovered from the stroke and is now taking Warfarin 21 mg/week as the oral anticoagulant therapy. Apart from Warfarin, he also takes Enalapril 10 mg/day, Aspirin 81 mg/day, Furosemide 20 mg/day, and Simvastatin 20 mg/day.

At the emergency department, his vital signs were all normal. Physical examination revealed irregular heart rhythm with valvular click sound at mitral and aortic valve area and engorged neck veins.

**Figure 1:**
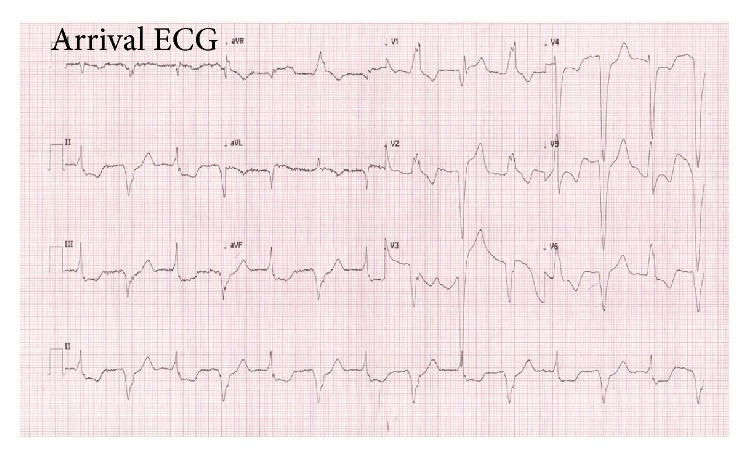
ECG recorded during the patient's arrival in emergency department showing atrial fibrillation with alternating right and left bundle branch block pattern with ST-T segment changes.

The patient was given a dose of 5 mg isosorbide dinitrate via sublingual route instantly but it did not improve his symptoms. His EKG revealed atrial fibrillation with regular ventricular rhythm of which morphologies of the QRS complexes alternated between a right bundle branch block (RBBB) and left bundle branch block (LBBB) pattern. This was highly suspicious of complete heart block at a level below atrioventricular nodal (infranodal block). Among RBBB morphology complexes (as shown in even beats of Figure 1), there was ST-segment elevation in leads V2, V3, I, and aVL with poor R progression in V4–V6. Also, there was reciprocal ST-segment depression in leads II, III, and aVF. These findings may indicate myocardial injury of the anterior and high lateral wall. Among LBBB morphology complexes (shown in odd beats of Figure 1), there were significant ST-segment changes in V4 and V5 (concordant leads) as well as ST-segment changes in V2, V3, II, III, and aVF (discordant leads), which fulfilled Sgarbossa criteria [8] and were compatible with a diagnosis of acute myocardial infarction (AMI) (Figure 1).

Cardiac biomarkers were all elevated. Creatine phosphokinase (CPK) was 6820 U/mL (normal range; 25–170 U/mL), creatine kinase MB isozyme (CK-MB) was 750 U/mL (normal range; 0–24 U/mL), and troponin-T (Trop-T) was >10,000 pg/mL (normal range; 13–25 pg/mL). Also, the coagulogram reported prolonged PT (PT = 19.2 seconds) and prolonged PTT (PTT = 29.6 seconds), and INR was in the subtherapeutic range (INR = 1.61). In addition, a chest X-ray demonstrated cardiomegaly with perihilar pulmonary congestion.

Based on these findings, the patient was diagnosed with AMI. A 325 mg dose of Aspirin, a 600 mg dose of Clopidogrel, and 5000 units of Heparin were given to the patient immediately at the emergency department. He received coronary angiography within 90 minutes of arrival. The coronary angiogram revealed 100% stenosis due to coronary emboli at mid part of left circumflex artery (LCx). Thrombectomy with an eliminate aspiration catheter (Terumo, Japan (N)) was successfully done and large red thrombi were removed from the arterial lumen. The underlying vessel structure was normal. Ball and cage prosthetic aortic valve and mitral valve appeared to be in a good condition (Figure 3).

**Figure 2:**
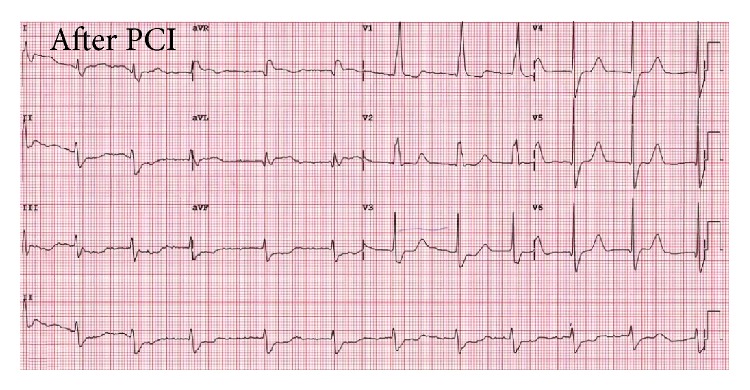
ECG after procedure demonstrated atrial fibrillation with complete right bundle branch block without Q wave revealed.

**Figure 3:**
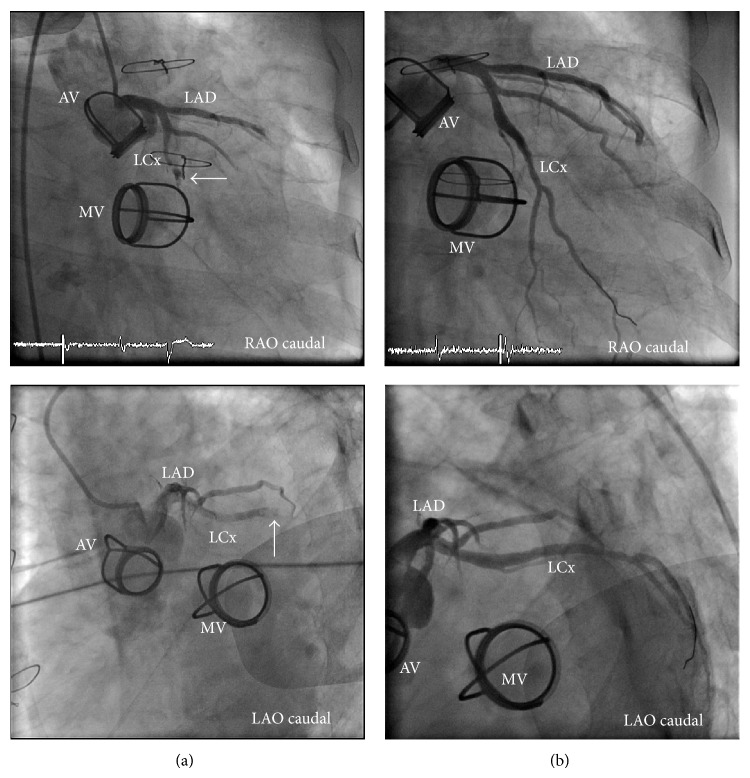
Angiogram of left coronary artery demonstrating filling defect (white arrow) at the mid portion of LCx (a). After thrombectomy with elimination aspiration catheter, blood flow in LCx was completely restored. The underlying vessel lumen appeared to be normal (b). ^*^LAD-left anterior descending artery, LCX-left circumflex artery, LAO-left anterior oblique, RAO-right anterior oblique, AV-aortic prosthetic valve, and MV-mitral prosthetic valve.

Transthoracic echocardiography (TTE), performed in the coronary care unit in the same day after cardiac catheterization, revealed global left ventricular hypokinesia with an ejection fraction of 32%, proper opening and closing functions of both ball valves with acceptable pressure gradients across both prostheses, no visible intracardiac thrombi, and intact interatrial septum. The patient had no symptoms of chest pain and his vital signs were stable. However, his EKG still showed atrial fibrillation with complete right bundle branch block (Figure 2). The treatment with intravenous unfractionated heparin was initiated for bridging therapy. The heparin dose was titrated until the optimal APTT level was reached. Warfarin dose was increased from 21 mg/week to 24 mg/week to attain therapeutic range of INR. Four days after the event, he was discharged home without any known complications.

## 3. Discussion

This is a case of a patient with a history of dual prosthetic heart valves and atrial fibrillation who developed AMI from coronary emboli due to inadequate anticoagulation. In spite of the rarity of coronary embolism, it should always be considered in the scenario of a prosthetic heart valve patient who presents with acute chest pain with a subtherapeutic INR.

Regarding the arrival ECG, while a LBBB pattern can obscure or mimic the diagnosis of AMI, Sgarbossa criteria can be a very useful tool for the AMI diagnosis in such circumstances [8]. Also, even if there are many causes of RBBB pattern in the ECG, AMI must always be considered.

Although coronary embolism from prosthetic valves is an uncommon cause of acute coronary syndrome, there are a number of previously reported occurrences. In 2008, Iakobishvili et al. [9] reported a series of 40 prosthetic heart valve cases who developed acute coronary syndrome. Only 2 from those 40 cases had prosthetic valve derived coronary emboli. In 2009, Sial et al. [10] reported a case of mitral valve prosthesis who developed AMI from coronary emboli and received successful management by clot extraction during coronary angiography and continued glycoprotein IIb/IIIa antagonist for 24 hours.

Coronary embolism usually involves the left coronary artery system more than the right. Within the left system, the left anterior descending artery is involved more than the left circumflex artery [11], while in the present case the embolism involves the mid portion of the left circumflex artery.

There is no definite guideline for the management of coronary emboli [12]. Many treatment options have been reported previously in the literature including coronary artery bypass graft, intracoronary thrombolysis [5], intravenous fibrinolytic therapy [13], and percutaneous intervention with clot removal [14]. Our patient was managed by coronary catheterization and thrombectomy with an aspiration catheter. After the procedure, the patient has no chest discomfort or any other symptoms and was finally discharged four days after admission. Apart from proper management, early recognition of the symptoms and early diagnosis are important factors contributing to the good prognosis.

Key learning points from our case include an interesting ECG pattern for a diagnosis of AMI lacking adequate anticoagulation and successful management by coronary angiography with clot removal.
